# Increases in United States life expectancy through reductions in injury-related death

**DOI:** 10.1186/s12963-017-0150-4

**Published:** 2017-08-30

**Authors:** Scott R. Kegler, Grant T. Baldwin, Rose A. Rudd, Michael F. Ballesteros

**Affiliations:** 1 Centers for Disease Control and Prevention, National Center for Injury Prevention and Control, Division of Analysis, Research, and Practice Integration, 4770 Buford Highway, Atlanta, GA 30341 USA; 2 Centers for Disease Control and Prevention, National Center for Injury Prevention and Control, Division of Unintentional Injury Prevention, 4770 Buford Highway, Atlanta, GA 30341 USA

**Keywords:** Life expectancy, Injury death, Cost of injury

## Abstract

**Background:**

During the previous century the average lifespan in the United States (US) increased by over 30 years, with much of this increase attributed to public health initiatives. This report examines further gains that might be achieved through reduced occurrence of injury-related death.

**Methods:**

US life tables and injury death rate data were used to estimate potential increases in life expectancy assuming various reductions in the rate of fatal injuries. Corresponding numbers of deaths potentially averted annually were also estimated; unit (per death) medical and lifetime work loss costs were employed to estimate total costs potentially averted annually.

**Results:**

Through elimination of injury as a cause of death, average US life expectancy at birth could be increased by approximately 1.5 years, with notable variations by sex, ethnicity, and race. More conservatively, average life expectancy at birth could be increased by 0.41 years assuming that the national injury death rate could be brought into line with the lowest state-specific rate. Under this more conservative but plausible assumption, an estimated 48,400 injury deaths and $61 billion in medical and work loss costs would be averted annually.

**Conclusions:**

Increases in life expectancy of the magnitude considered in this report are arguably attainable based on long-term historical reductions in the US injury death rate, as well as significant continuing reductions seen in other developed countries. Contemporary evidence-based interventions can play an important role in reducing injury-related deaths, such as those due to drug overdoses and older adult falls, as well as suicides.

## Background

Injuries represent a major public health concern in the United States and are the leading cause of death for individuals 1–44 years of age [1]. Each year, fatal and nonfatal injuries result in approximately $670 billion in combined medical and work loss costs for the nation [2]. Implementation of effective interventions can help prevent injuries and the attendant social and economic burdens [3, 4].

The average lifespan in the US increased by over 30 years during the previous century, with much of the increase attributed to various public health interventions [5, 6]. Despite such gains, the US lags behind other developed countries in terms of continuing improvements in population health [7]. For example, the US ranks near the bottom among 34 member countries in the Organisation for Economic Co-operation and Development (OECD) in life expectancy and in years of life lost due to premature mortality for most causes of death, and in particular for injury-related causes [7].

Many injuries are predictable and preventable, resulting in needless disability and death [8]. As a point of reference, the Vision Zero road safety initiative in Sweden is centered on the premise that no loss of life is acceptable while simultaneously recognizing that human error is inevitable [9]. Correspondingly, Sweden designed a road safety system – vehicles, roads, and laws that govern driving behavior – providing multiple layers of protection [9]. Sweden has among the lowest motor vehicle fatality rates in the world because of this ambitious commitment to injury prevention [10]. With respect to injury deaths due to intentional self-harm (suicide) or interpersonal violence (homicide), reported rates for certain other OECD countries are both low relative to US rates. For the United Kingdom, Italy, and Spain, for example, suicide rates reported for the year 2012 were approximately half the US rate, and homicide rates for the same year were just a small fraction of the US rate [11]. Whether attributable to structural, cultural, legal, or other differences, injury-related death rates in these and other OECD countries suggest the potential for substantial improvements in the US.

Life expectancy at birth, the average duration that children in a given birth cohort (e.g., those born in 2012) are expected to live from the date of birth assuming prevailing age-specific death rates [12], is a particularly useful measure as it is readily interpreted and can be used to demonstrate and quantify improvements in population health. Beyond public health professionals and researchers, life expectancy can be understood by legislators responsible for the laws and regulations that influence health policy and practice, as well as by the general public.

To help understand how potential future reductions in the injury-related death rate could impact population health in the US, this report presents two main analyses. First, projected increases in US life expectancy are heuristically estimated assuming various reductions in injury death rates, ranging from complete elimination of injury death to more conservative reductions. Because injury death rates vary substantially by sex and ethnicity/race, selected results are presented by these factors in order to illustrate the potential for impacting specific subpopulations. Second, several companion measures are calculated, characterizing the numbers of deaths and associated costs that might be averted through such reductions.

The objectives of the current study overlap to some extent with those of an earlier study [13] that explored gains in life expectancy assuming complete elimination of selected causes of death including injury, and relying on more detailed methodology than the heuristic approach applied here. As outlined above, however, the objectives of the current study extend further to include estimation of gains associated with less than complete elimination of injury-related death and accompanying reductions in the cost burden.

## Methods

### Data

The procedure for estimating increases in life expectancy relies on National Center for Health Statistics (NCHS) US life tables for the year 2012 (the most recent available at the time of this analysis) and annualized US injury death rates (per 100,000 resident population) based on NCHS vital statistics data for the period 2011–2013. For a given population group (e.g., males) the corresponding life table shows the estimated remaining life expectancy at each age from 0 to 99 years, terminating with a composite estimate for 100 years of age and older [12]. The year 2012 life tables are stratified by sex, Hispanic ethnicity, race (white or black only), and combinations thereof. Age-specific injury death rates similarly stratified by sex, Hispanic ethnicity, and race were retrieved using the CDC WISQARS reporting application [1]. In WISQARS, injury deaths are identified based on the single underlying-cause-of-death code in each death record, which must indicate injury. Additional cause/condition codes listed in the death records do not contribute to the identification of injury deaths. Rates were downloaded for all injury deaths combined (*International Classification of Diseases, 10th Revision* [ICD-10] underlying cause codes V01–Y36, Y85–Y87, Y89, *U01–*U03); and separately for unintentional injury deaths (V01–X59, Y85–Y86), which most notably include unintentional poisonings involving drug and non-drug agents, motor vehicle crashes, and falls; and separately for violence-related injury deaths (X60–Y09, Y35, Y87.0–Y87.1, Y89.0, *U01–*U03), which primarily include suicides and homicides. To obtain adequate precision by single year of age, annualized rates were tabulated for the composite three-year period (2011–2013) centered about the year 2012.

Estimation of deaths and costs averted further requires US population estimates by single year of age, as well as unit (per injury death) medical and work loss cost estimates, also by single year of age. Age-specific resident population estimates for calendar year 2012 (coinciding with the reference year for the life expectancy calculations) and age-specific unit medical and work loss cost estimates in year 2015 dollars (the most recent available at the time of this analysis) were similarly retrieved using the CDC WISQARS reporting application [1]. For fatal injuries, unit medical cost estimates reflect coroner/medical examiner costs, emergency transport costs, emergency department costs, and hospital/nursing home/hospice costs [14]. The unit medical cost estimate assigned to each decedent record depends on the mechanism of injury, place of death, and decedent age [1]. Unit work loss cost estimates reflect projected lost earnings and the projected value of lost benefits and self-provided household services that would have accrued over a decedent’s expected remaining lifetime [14]. The unit work loss cost estimate assigned to each decedent record depends on decedent sex and age [1]. Average (per decedent) unit medical and work loss costs expressed in year 2015 dollars, by single year of age, were calculated and downloaded from WISQARS for all injury deaths combined, and also separately for unintentional injury deaths and violence-related injury deaths.

### Estimation approach

The heuristic approach to estimating increases in life expectancy associated with complete elimination of injury-related death involves revising existing life tables in four steps. Although these steps do not encompass all of the details involved in the construction of the original life tables, the intent here is to capture enough detail to reasonably estimate increases in life expectancy.

For a given life table, the first step involves abridging the terminal age interval to 85 years of age and older. This is done to facilitate the subsequent integration of injury death rates, which are not available by single year of age beyond 84 years through either the CDC WISQARS or CDC WONDER reporting applications [1, 15]. The process is straightforward and adheres to the abridgement method specified in the NCHS technical report describing the 2012 life tables [12]. Details and an example are provided in the technical Appendix to this manuscript. Notably, this first step leaves estimated life expectancy at each year of age (including at birth) unchanged.

The second step involves revising downward the life table death rate for each one-year age interval from 0 to 1 years through 84–85 years, to reflect the hypothetical exclusion of injury as a cause. This is done by subtracting the national injury-related death rate (per unit population) for each year of age, adjusted for unstated decedent age and ethnicity/race misclassification [12], from the corresponding all-cause death rate determined from the life table entries. That is, the rate of non-injury death for the one-year age interval *x* to *x* + 1 years is estimated as:

$$ {rate_{ages\ x\  to\ x+ 1}}^{non\text{-}injury}\kern0.5em =\kern0.5em {rate_{ages\ x\  to\ x+ 1}}^{all\text{-}cause}-\kern0.5em {rate_{ages\ x\  to\ x+ 1}}^{injury\ (adjusted)}. $$

Of note, individuals for whom injury death is now assumed averted would return to the population (or cohort) at risk of death due to non-injury causes, implying that the estimated rate of non-injury death is negatively biased. However, a sensitivity analysis (not shown) suggests that such bias is very minimal, and as such no correction is attempted. The non-injury rate calculation is not needed for the terminal age interval. Further details are provided in the technical Appendix.

The third step employs the revised life table death rates to estimate survivorship (the number alive at the beginning of an age interval), deaths, and person-years lived for each age interval, beginning with an assumed cohort of 100,000 live births [12]. For the initial age interval (0–1 years), estimation of person-years lived incorporates the appropriate separation factor *f* reflecting a tendency for infant death to occur in the earlier part of the interval. For the terminal age interval (85 years and older), person-years lived is estimated by conservatively adopting the remaining years of life expectancy from the original life table (which includes the possibility of injury-related death), and applying it to the count of survivors entering the interval. The equations used to carry out these calculations are provided in the technical Appendix, along with an example illustrating the application of the separation factor.

As a fourth and final step, the revised life table entries resulting from the first three steps are summarized following documented life table methods [12], completing the revised life table. Details are provided in the technical Appendix. For the present investigation, revised life expectancy at birth is of primary interest. The difference between this estimate and life expectancy at birth from the original table represents the estimated gain in life expectancy at birth, after excluding injury as a cause of death.

Reflecting goals that may be more immediately achievable, the approach described above can also be applied to estimate increases in life expectancy associated with assumed reductions in injury-related death less far-reaching than elimination of all such deaths. Increases in life expectancy might, for example, be re-estimated under the assumption that the national injury-related death rate can be reduced by some specified fraction *r* (e.g., *r* = 0.25 corresponds to a 25% reduction). Again referring to adjusted national rates of injury death by single year of age to revise downward original life table death rates, the death rate assuming reduced-injury for the one-year age interval *x* to *x* + 1 years is estimated as:

$$ {rate_{ages\ x\  to\ x+ 1}}^{reduced\ injury}\kern0.5em =\kern0.5em {rate_{ages\ x\  to\ x+ 1}}^{all\text{-}cause}-\kern0.5em r\times {rate_{ages\ x\  to\ x+ 1}}^{injury\ (adjusted)}. $$

Reductions in the age-specific rates of injury-related death represented by the rightmost term above can be applied to national population figures by year of age, in order to estimate age-specific (and total) deaths averted due to any hypothetical reduction in injury-related death. Denoting the size of the general population of age *x* by *P*_*x*_, the estimated reduction in the number of injury deaths for the one-year age interval *x* to *x* + 1 years would be given by:

$$ {R}_x\kern0.5em =\kern0.5em r\times {rate_{ages\ x\  to\ x+ 1}}^{injury\ (adjusted)}\times {P}_x. $$

Estimated age-specific reductions in injury-related death can further be coupled with age-specific unit medical and work loss costs to arrive at corresponding estimates of costs averted. In this context, it should be understood that it is the injury-producing events that are hypothetically being prevented or significantly mitigated, as opposed to shifting injury outcomes from fatal to nonfatal through such measures as improved trauma care. Denoting the average combined unit medical and work loss costs across all injury deaths occurring at age *x* in the general population by *C*_*x*_, estimated costs averted for the corresponding one-year age interval *x* to *x* + 1 years would be given by:

$$ {A}_x={R}_x\times {C}_x. $$

The proposed methods can also be applied to estimate increases in life expectancy, deaths averted, and costs averted under assumed reductions in specific injury categories. In the present analysis, reductions in unintentional injury deaths and violence-related deaths were additionally considered.

## Results

Table 1 shows the original life expectancy at birth (reflecting all causes of death) and the revised life expectancy at birth assuming complete elimination of injury-related death for the general population, followed by estimates stratified by sex, by ethnicity/race, and by ethnicity/race/sex. Injury death rates are also shown for each population group as supplementary information [1].

**Table 1:** Estimated increases in life expectancy assuming elimination of injury-related death

Population group	Injury death rate 2012	Life expectancy for 2012 birth cohort		Estimated increase
		Original estimate for all causes	Revised estimate without injury	
All persons	60.6 per 100,000	78.8 years	80.3 years	1.5 years
Male	83.1 per 100,000	76.4 years	78.4 years	2.0 years
Female	38.8 per 100,000	81.2 years	82.0 years	0.8 years
Non-Hispanic white	70.8 per 100,000	78.9 years	80.4 years	1.5 years
Non-Hispanic black	58.3 per 100,000	75.1 years	76.7 years	1.6 years
Hispanic	33.0 per 100,000	81.9 years	83.0 years	1.1 years
Non-Hispanic white male	93.7 per 100,000	76.5 years	78.6 years	2.1 years
Non-Hispanic black male	92.1 per 100,000	71.9 years	74.2 years	2.3 years
Hispanic male	49.1 per 100,000	79.3 years	80.8 years	1.5 years
Non-Hispanic white female	48.6 per 100,000	81.2 years	82.2 years	1.0 years
Non-Hispanic black female	27.3 per 100,000	78.1 years	78.9 years	0.8 years
Hispanic female	16.4 per 100,000	84.3 years	84.9 years	0.6 years

As might have been anticipated, the results indicate that population subgroups with higher injury death rates tend to show greater potential for gains in life expectancy through the elimination of injury as a cause of death. The results by sex suggest that the gap in life expectancy between males and females might be substantially reduced; potential reductions in the gaps by ethnicity/race are less notable.

**Fig. 1:**
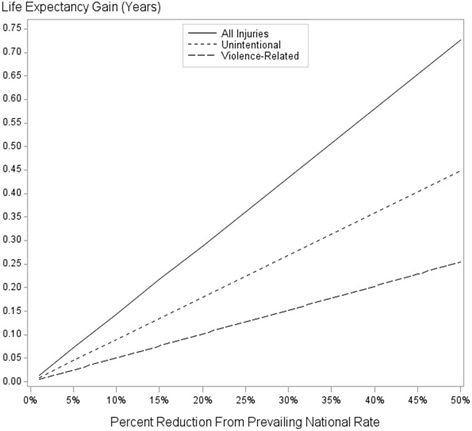
Estimated increases in life expectancy assuming various reductions in injury-related death

Figure 1 shows the estimated gains in life expectancy at birth for the general population, alternatively assuming various partial reductions (ranging up to 50%) in prevailing national injury-related death rates. Separate lines are shown for all injury-related deaths, unintentional injury deaths, and violence-related deaths. A reduction of 50% in prevailing national rates corresponds to respective gains in life expectancy of 0.73 years (all injury-related deaths), 0.45 years (unintentional injury deaths), and 0.25 years (violence-related deaths).

**Fig. 2:**
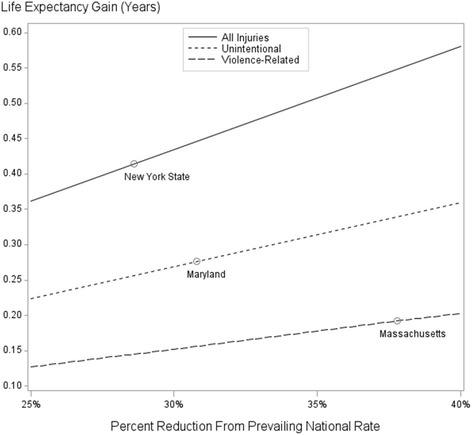
Estimated increases in life expectancy assuming specific reductions in injury-related death

During the period covered by this analysis, the lowest state-specific annualized rate for all-cause injury deaths was 28.6% below the national rate (43.23 per 100,000 population in New York State compared to 60.58 per 100,000 nationally) [1]. For unintentional injury deaths the lowest state-specific rate was 30.8% below the national rate (28.28 per 100,000 in Maryland compared to 40.84 per 100,000 nationally) and for violence-related deaths the lowest state-specific rate was 37.8% below the national rate (11.34 per 100,000 in Massachusetts compared to 18.24 per 100,000 nationally) [1]. Figure 2 illustrates the estimated gains in life expectancy at birth for the general population, assuming that the national rate is brought into line with the lowest state-specific rate in each injury category. Under these more conservative reductions, the respective gains in life expectancy are 0.41 years (all injury-related deaths), 0.28 years (unintentional injury deaths), and 0.19 years (violence-related deaths). It should be noted that because the lowest state-specific rates across injury categories were observed for three different states, the sum of the estimated gains in life expectancy for the assumed reductions in unintentional injury death and violence-related death can (and here does) exceed the estimated gain in life expectancy for the assumed reduction in all-cause injury death (the latter representing the smallest percentage reduction).

Under the assumed percentage reduction in all-cause injury death shown in Fig. 2, approximately 48,400 deaths would have been averted in 2012, representing $61.4B (in year 2015 dollars) in averted medical and work loss costs. For the assumed reduction in unintentional injury death, approximately 33,400 deaths would have been averted, representing $39.6B in averted costs; for the assumed reduction in violence-related death, approximately 21,200 deaths would have been averted, representing $30.2B in averted costs. Here again it should be noted that because the lowest state-specific rates across injury categories were observed for different states, estimated deaths averted and estimated costs averted for the unintentional and violence-related injury categories combined can (and do) exceed estimated deaths averted and estimated costs averted for the all-cause injury category.

## Discussion

### Comparison of estimates between studies

The heuristic estimates derived under the assumption of complete elimination of injury death agree quite closely with estimates from the earlier study exploring gains in life expectancy based on data for 1999–2001 [13]. For example, the earlier study concluded that the gain in life expectancy at birth in the general population, assuming elimination of unintentional injury death, would be 0.84 years; the gain assuming elimination of death due to self-harm (suicide) would be 0.26 years; and the gain assuming elimination of assault-related death (homicide) would be 0.20 years [13]. While not strictly additive, the gains in life expectancy across these three categories, which historically represent nearly all deaths due to injury [1], sum to 1.3 years. The current study finds an estimated gain in life expectancy at birth in the general population, assuming elimination of all injury deaths, of 1.5 years. Similar agreement between the two studies holds within population subgroups, for example for males (1.8 years life expectancy gain in the earlier study [13] compared to 2.0 years in the current study) and females (0.8 years life expectancy gain in the earlier study [13] compared to 0.8 years in the current study).

### Magnitude and impact of life expectancy increases

An estimated increase in life expectancy at birth of approximately 1.5 years for the overall population, assuming complete elimination of injury as a cause of death, is significant because it applies to entire birth cohorts (e.g., numbering nearly 4 million in the US for the year 2012 [16]). Yet given that injury is the leading cause of death among US residents in the 1–44 year age group, the question arises as to why the estimated increase is not larger. There are two apparent reasons.

First, the fraction of all deaths occurring in the 1–44 year age group is relatively small. During the year 2012, there were approximately 2.54 million deaths among US residents of all ages, with approximately 152,400 of these deaths (6.0%) occurring in the 1–44 year age group [15]. Within the 1–44 year age group, injury was the underlying cause in approximately 78,800 (51.7%) of all deaths [15]. Thus, injury was not just the leading cause of death in this age group, it was the predominant cause, outweighing all other causes combined. It might therefore seem that eliminating injury as a cause of death holds great potential for increasing life expectancy. But because there are relatively few deaths in this age group due to any cause, the potential is limited. Hypothetically eliminating the predominant cause of death within a small subset of all deaths (i.e., those occurring in the 1–44 year age group) does not translate to a dramatic increase in estimated life expectancy.

Second, and notwithstanding the fact that injury is the predominant cause of death in the 1–44 year age group, more injury deaths occur among persons 45 years of age and older. Even so, injury causes only a small fraction of all deaths in this older group. During 2012, there were approximately 2.37 million deaths among US residents 45 years of age or older, with approximately 110,000 of these deaths (4.6%) being due to injury [15]. Hypothetically eliminating a comparatively minor cause of death within a large subset of all deaths (i.e., those occurring in the population 45 years of age and older) again does not translate to a dramatic increase in estimated life expectancy.

Alternatively considering more immediately achievable reductions in injury-related death, such as bringing the national all-cause injury death rate into line with the lowest observed state-specific rate, the estimated life expectancy gain is notably smaller. This more modest gain nevertheless translates to tens-of-thousands of deaths and tens-of-billions of dollars in costs that might be averted each year. Of note, the estimate of unintentional injury deaths that might be averted annually by bringing the national rate of unintentional injury death into line with the lowest observed state-specific rate is reasonably consistent with estimates derived using an alternate analytical approach [17].

### Limitations

While key aspects of the original life table methodology [12] have been incorporated into the heuristic approach applied in the present analysis, some of the more complex aspects of the original methodology were not replicated. Further, adoption of estimated life expectancy for the terminal age interval considered here (i.e., 85 years and older) from the original life tables (which incorporate all causes of death including injury), likely resulted in modest underestimation of increases in life expectancy at birth, deaths averted, and medical and work loss costs averted. In this regard, the estimates presented here might thus be considered somewhat conservative. Similarly, the heuristic approach applied here is much less complex than the approach employed in the earlier study [13] examining gains in life expectancy associated with elimination of selected causes of death. Despite its lack of complexity, however, the heuristic approach resulted in estimated gains in life expectancy quite comparable (as quantified above) to those of the earlier study.

Work loss costs typically represent the major component of total (combined medical and work loss) costs associated with injury-related deaths. In applying unit work loss costs to estimated numbers of deaths averted, it is implicitly assumed that hypothetical survivors would generally represent typical members of their age cohort in terms of future risks and life expectancy. While not quantifiable from the available data, to whatever extent that survivors might be atypical the estimates of averted costs could be correspondingly distorted. Another recent study of differences in life expectancy across developed countries involved a similar (and untested) assumption that the risk of death due to non-injury causes would remain unaffected after the exclusion of selected major causes of injury-related death [18].

## Conclusions

Increases in life expectancy of the magnitude considered in this report are arguably attainable based on historical reductions in the US injury death rate. For example, during the previous century the rate of death (age-adjusted) from unintentional injuries decreased by over 60% in the US [19]. An important component of this decrease was the progress made in reducing motor vehicle traffic (MVT) deaths. Beginning about 1980 and continuing through the first decade of the current century, annual MVT deaths in the US declined from approximately 51,000 to approximately 33,000 – a decrease of over one-third [20]. This decrease occurred despite the fact that over the same period the yearly number of vehicle-miles traveled nearly doubled, from approximately 1.5 trillion to just under 3 trillion [20]. These gains were achieved through improvements in vehicle and roadway designs, as well as stricter and more widespread traffic safety laws such as those pertaining to intoxicated driving and those mandating the use of personal protective equipment including safety belts, child safety seats, and motorcycle helmets [21].

Notwithstanding long-term historical improvements, injury remains a persistent and evolving problem in the US. From 2000 to 2015, the national age-adjusted injury death rate increased by approximately 20% – from 52.75 per 100,000 population to 63.65 per 100,000 population [1]. This increase is primarily attributable to increases in deaths due to unintentional drug overdoses and older adult falls, and suicides, which currently represent three of the top four leading categories of injury death in the US [1]. The unintentional drug overdose death rate more than tripled between 2000 and 2015 (from 4.16 per 100,000 population to 13.77 per 100,000 population [age-adjusted]), due largely to increases in deaths from opioid pain medications [1, 22]; the rate of death due to unintentional falls among older adults (65 years and older) doubled over this same period (from 29.53 per 100,000 to 60.55 per 100,000 [age-adjusted]) [1]; and the rate of suicide (among persons 10 years and older) increased by approximately one-fourth over this period (from 12.16 per 100,000 to 15.44 per 100,000 [age-adjusted]) [1].

Broad implementation of contemporary evidence-based interventions can play an important role in efforts to reduce the social and economic burdens associated with injuries [23]. As an example, a coordinated, multisector, and multifaceted effort addressing the drug overdose problem is underway at the state level. This includes improving clinical practices leading to safer prescribing of opioid pain relievers, expanding the use of medication-assisted treatment for persons with opioid use disorder, and increasing access to naloxone – a lifesaving drug that can counteract the effects of overdoses [24]. Following efforts in one state to improve prescribing practices, the drug overdose death rate (all intents) fell by nearly 18% [25]. Evidence-based interventions directed at older adult fall prevention and suicide prevention can similarly contribute to reducing the burdens associated with injury-related death [26, 27]. Given the decreases in injury morbidity and mortality in other developed countries, it is reasonable to expect that future improvements are possible in the US [28, 29].

## Appendix

### Life table age intervals

The NCHS life table age intervals are indexed by the subscript *x* [12]. For one-year age intervals, *x* refers to ages *x* to *x* + 1 years. For the terminal age interval, *x* refers to ages *x* years and older.

### Abridging the terminal age interval

The NCHS life table abridgement procedure [12] is applied in the present study to abridge the terminal age interval in a given life table to 85 years and older. Beginning with an original NCHS life table [12] having a terminal age interval of 100 years and older (Table 2), the lines for age intervals 86–87 years, …, 100 years and older are deleted. The new terminal age interval is 85 years and older. The probability of dying (*q*_*x*_) in the new terminal interval is set to 1.0, the number of persons dying (*d*_*x*_) is set equal to the number of persons surviving to beginning of the interval (*l*_*x*_), and person-years lived during the interval (*L*_*x*_) is set equal to the number of person-years lived after the beginning of the interval (*T*_*x*_) [12]. The remaining rows of the table are not affected, nor is the estimated life expectancy column (*e*_*x*_) (Table 3).

**Table 2:** Life table for the total population: United States, 2012

Age interval *x* to *x* + 1 years	Probability of dying between ages *x* and *x* + 1	Number surviving to age *x*	Number dying between ages *x* and *x* + 1	Person-years lived between ages *x* and *x* + 1	Total number of person-years lived above age *x*	Expectation of life at age *x*
	*q*_*x*_	*l*_*x*_	*d*_*x*_	*L*_*x*_	*T*_*x*_	*e*_*x*_
0–1	0.005978	100,000	598	99,474	7,882,683	78.8
1–2	0.000409	99,402	41	99,382	7,783,209	78.3
2–3	0.000270	99,362	27	99,348	7,683,827	77.3
▪	▪	▪	▪	▪	▪	▪
▪	▪	▪	▪	▪	▪	▪
▪	▪	▪	▪	▪	▪	▪
84–85	0.074785	45,578	3409	43,874	320,902	7.0
85–86	0.083577	42,169	3524	40,407	277,029	6.6
86–87	0.093319	38,645	3606	36,842	236,621	6.1
▪	▪	▪	▪	▪	▪	▪
▪	▪	▪	▪	▪	▪	▪
▪	▪	▪	▪	▪	▪	▪
98–99	0.285296	4008	1144	3436	10,386	2.6
99–100	0.306203	2865	877	2426	6949	2.4
100 and over	1.000000	1987	1987	4523	4523	2.3

Source: *NCHS* National Vital Statistics System, Mortality

**Table 3:** Abridged life table for the total population: United States, 2012

Age interval *x* to *x* + 1 years	Probability of dying between ages *x* and *x* + 1	Number surviving to age *x*	Number dying between ages *x* and *x* + 1	Person-years lived between ages *x* and *x* + 1	Total number of person-years lived above age *x*	Expectation of life at age *x*
	*q*_*x*_	*l*_*x*_	*d*_*x*_	*L*_*x*_	*T*_*x*_	*e*_*x*_
0–1	0.005978	100,000	598	99,474	7,882,683	78.8
1–2	0.000409	99,402	41	99,382	7,783,209	78.3
2–3	0.000270	99,362	27	99,348	7,683,827	77.3
▪	▪	▪	▪	▪	▪	▪
▪	▪	▪	▪	▪	▪	▪
▪	▪	▪	▪	▪	▪	▪
84–85	0.074785	45,578	3409	43,874	320,902	7.0
85 and over	1.000000	42,169	42,169	277,029	277,029	6.6

Source: *NCHS* National Vital Statistics System, Mortality

### Estimating death rates excluding injury as a cause

All-cause death rates (*m*_*x*_) are not shown in the NCHS life tables, but can be reconstructed for each one-year age interval, by dividing the indicated number dying in the interval by the indicated number of person-years lived during the interval (*m*_*x*_ = *d*_*x*_ / *L*_*x*_) [12].

Annualized injury death rates (per 100,000 population) by single year of age obtained from the WISQARS Fatal Injury Reports application [1] were divided by 100,000 to express them on a per unit population scale (*m*_*x*_^*injury*^). These rates were then adjusted slightly upward to account for a small number of decedents with unstated age. For rates representing subpopulations by ethnicity/race, adjustments were also made for ethnicity/race misclassification by applying the indicated all-ages classification ratio from the NCHS life table report [12]; for the overall population (and the overall population stratified by sex) the classification ratio is taken to equal unity. Combining both adjustments, the adjusted injury death rate between ages *x* and *x* + 1 years is given by:

$$ {m_x}^{injury\ adjusted}\kern0.5em =\kern0.5em {m_x}^{injury}\times \left( total\ injury\ deaths/ injury\ deaths\ with\  age\  stated\right)\times \kern0.5em classification\ ratio. $$

Of note, adjustments for unstated age and ethnicity/race misclassification are already incorporated into the original life table components.

The rate of non-injury death between ages *x* and *x* + 1 years is then estimated by subtraction:

$$ {m_x}^{non\text{-}injury}\kern0.5em =\kern0.5em {m}_x\kern0.5em -\kern0.5em {m_x}^{injury\ adjusted}. $$

This sequence of calculations is not needed for the terminal age interval.

### Estimating survivorship, deaths, and person-years lived for the revised life table

For each one-year age interval, the probability of dying due to non-injury causes is calculated as [12]:

$$ {q_x}^{non\text{-}injury}\kern0.5em =\kern0.5em {m_x}^{non\text{-}injury}/\left(1\kern0.5em +\kern0.5em \left(1-{a}_x\right)\times {m_x}^{non\text{-}injury}\right). $$

The term *a*_*x*_ reflects the average time lived during an interval by individuals dying within it [12]. For the initial age interval (0–1 years) the heuristic estimation approach employs the shortcut assumption that *a*_*0*_ = *f*, the indicated separation factor (more on separation factors below) for the given population group. Empirically, this introduces very minor distortion. For the subsequent one-year age intervals, *a*_*x*_ = ½, adhering to the formal life table approach [12]. For the terminal age interval, the probability of death *q*_*85*_^*non-injury*^ = 1.0, consistent with the usual life table approach [12].

The number surviving to the beginning of the initial age interval *l*_*0*_^*non-injury*^ = 100,000 represents the assumed starting live birth cohort [12]. For each subsequent age interval, including the terminal age interval, the number surviving to the beginning of the interval is estimated by multiplying the number alive at the beginning of the prior interval by the probability of surviving through that interval [12]:

$$ {l_x}^{non\text{-}injury}\kern0.5em =\kern0.5em {l_{x- 1}}^{non\text{-}injury}\times \left(1-{q_{x- 1}}^{non\text{-}injury}\right). $$

The number dying in each age interval is estimated by multiplying the number alive at the beginning of the interval by the probability of death during the interval [12]:

$$ {d_x}^{non\text{-}injury}\kern0.5em =\kern0.5em {l_x}^{non\text{-}injury}\times {q_x}^{non\text{-}injury}. $$

Estimation of person-years lived during the initial one-year age interval incorporates a separation factor *f*, reflecting a tendency for death to occur in the earlier part of this interval [12]. The separation factor is specific to the given population group. As an example, the separation factor for the overall population is *f* = 0.120 [12], and is applied as follows, giving smaller weight to the number surviving to the beginning of the initial age interval (i.e., the entire birth cohort) and greater weight to the number surviving to the beginning of the next age interval [12]:

$$ {L_0}^{non\text{-}injury}\kern0.5em =\kern0.5em f\times {l_0}^{non\text{-}injury}\kern0.5em +\kern0.5em \left(1-f\right)\times {l_1}^{non\text{-}injury}. $$

For each subsequent one-year age interval, estimated person-years lived reflects the assumption that deaths occur across the interval with uniform likelihood [12]:

$$ {L_x}^{non\text{-}injury}\kern0.5em =\kern0.5em \frac{1}{2}\times {l_x}^{non\text{-}injury}\kern0.5em +\kern0.5em \frac{1}{2}\times {l_{x+ 1}}^{non\text{-}injury} $$

which, based on the earlier expressions for *l*_*x*_^*non-injury*^ and *d*_*x*_^*non-injury*^, simplifies to [12]:

$$ {L_x}^{non\text{-}injury}\kern0.5em =\kern0.5em {l_x}^{non\text{-}injury}\kern0.5em -\kern0.5em \frac{1}{2}\times {d_x}^{non\text{-}injury}. $$

Estimating person-years lived for the terminal age interval is more complicated because the terminal interval is open-ended – an individual surviving to the beginning of the interval might live an arbitrary number of additional years. For the terminal age interval considered here (85 years and older), injury deaths constitute a very small fraction of all deaths (under 3%) occurring in the general population [15]. The remaining years of life expectancy from the original life table, *e*_*85*_, should thus closely approximate *e*_*85*_^*non-injury*^. Noting that *e*_*x*_ = *T*_*x*_ / *l*_*x*_ in general and that *L*_*x*_ = *T*_*x*_ for the terminal age interval [12], *e*_*85*_ is adopted as a surrogate for *e*_*85*_^*non-injury*^ and applied to the count of survivors entering the terminal interval to estimate person-years lived during it. This approach is conservative, in that *e*_*85*_ ≤ *e*_*85*_^*non-injury*^, so any estimation bias in remaining person-years lived will be downward. As an example, for the overall population, it would be assumed that *e*_*85*_^*non-injury*^ = *e*_*85*_ ≅ 6.6 years (see the abridged life table above), and person-years lived during the terminal age interval would be estimated by:

$$ {L_{85}}^{non\text{-}injury}\kern0.5em =\kern0.5em {e_{85}}^{non\text{-}injury}\times {l_{85}}^{non\text{-}injury}. $$

### Completing the revised life table

The remainder of the process for completing a revised life table is straightforward. For each age interval, including the terminal age interval, the total number of person-years lived past the starting age (*x*) of the interval is given by [12]:

$$ {T_x}^{non\text{-}injury}=\sum \limits_{k=x}^{85}{L_k}^{non\text{-}injury}. $$

Finally, the estimated life expectancy from the starting age (*x*) for each one-year age interval is given by [12]:

$$ {e_x}^{non\text{-}injury}\kern0.5em =\kern0.5em {T_x}^{non\text{-}injury}/{l_x}^{non\text{-}injury}. $$

Estimated life expectancy for the terminal age interval, *e*_*85*_^*non-injury*^, was conservatively assumed equal to the estimated life expectancy from the original life table, *e*_*85*_, in a prior step.

## Abbreviations

ICD-10: International Classification of Diseases, 10th Revision
NCHS: National Center for Health Statistics
OECD: Organisation for Economic Co-operation and Development
